# Supplementary material for: In Vivo Neuroprotective Effects of Alpinetin Against Experimental Ischemic Stroke Damage Through Antioxidant and Anti-Inflammatory Mechanisms
Source: Int J Mol Sci. 2025 May 26;26(11):5093. doi: 10.3390/ijms26115093 (PMC12154397; doi:10.3390/ijms26115093)
Supplement: Supplementary file 1 [file ijms-26-05093-s001.zip › ijms-3661883-supplementary.pdf]

The HPLC analysis

Empower 3 SOFTWARE

Chengdu Biopurify

SAMPLE INFORMATION

Sample Name:	Alpinetin PRF23031622	Acquired By:	prf01
Sample Type:	Unknown	Sample Set Name	
Vial:	52	Acq. Method Set:	Alpinetin
Injection #:	1	Processing Method	sample
Injection Volume:	10.00 ul	Channel Name:	PDA Ch1 300nm@4.8nm
Run Time:	25.0 Minutes	Proc. Chnl. Descr.:	PDA Ch1 300nm@4.8nm
Date Acquired:	3/16/2023 10:46:11 AM CST		
Date Processed:	3/16/2023 11:11:57 AM CST		

Auto-Scaled Chromatogram

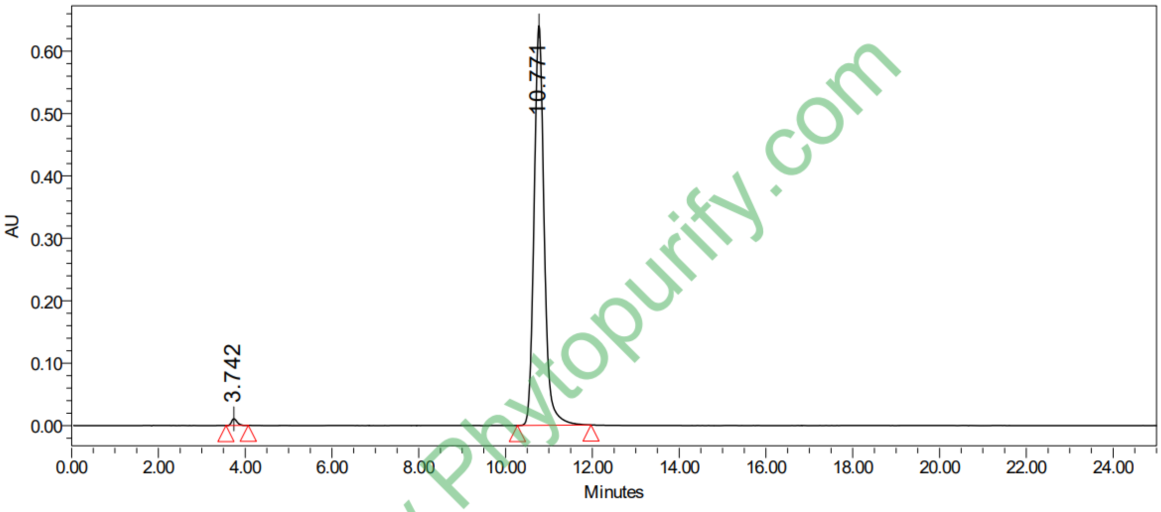

www.Phytopurify.com

Peak Results

	Name	RT	Area	% Area	Height (µV)	USP Plate Count	USP Resolution
1		3.742	95553	0.91	10726	4160.06	
2		10.771	10350178	99.09	640122	11131.55	21.50
